# Lung ultrasound assessment of atelectasis following different anesthesia induction techniques in pediatric patients: a propensity score-matched, observational study

**DOI:** 10.1186/s44158-024-00206-x

**Published:** 2024-10-05

**Authors:** Anna Camporesi, Giulia Roveri, Luigi Vetrugno, Danilo Buonsenso, Valentina De Giorgis, Sara Costanzo, Ugo Maria Pierucci, Gloria Pelizzo

**Affiliations:** 1 Department of Pediatric Anesthesia and Intensive Care, Buzzi Children’s Hospital, Via Castelvetro 32, 20154 Milan, Italy; 2 Department of Anesthesia and Intensive Care Medicine “F. Tappeiner” Hospital, Merano, Italy; 3 Eurac Research, Institute of Mountain Emergency Medicine, 39100 Bolzano, Italy; 4 Department of Medical, Oral and Biotechnological Sciences, University of Chieti-Pescara, Chieti, Italy; 5 Department of Woman and Child Health and Public Health, Fondazione Policlinico Universitario “A. Gemelli”, Rome, Italy; 6 Centro Di Salute Globale, Università Cattolica del Sacro Cuore, Rome, Italy; 7 Pediatric Surgery Department, Buzzi Children’s Hospital, Milan, Italy; 8 Department of Biomedical and Clinical Science, Luigi Sacco University Hospital, Milan, Italy

**Keywords:** Atelectasis, Pediatric anesthesia, Inhalatory induction, Intravenous induction, Lung ultrasound

## Abstract

**Introduction:**

Atelectasis is a well-documented complication in pediatric patients undergoing general anesthesia. Its incidence varies significantly based on surgical procedures and anesthesia techniques. Inhalation induction, commonly used to avoid the discomfort of venipuncture, is suspected to cause higher rates of respiratory complications, including atelectasis, compared to intravenous induction. This study aimed to evaluate the impact of inhalation versus intravenous anesthesia induction on atelectasis formation in pediatric patients, as assessed by lung ultrasound (LUS).

**Methods:**

This propensity score-matched observational study was conducted at a tertiary pediatric hospital in Milan, Italy. Inclusion criteria were children ≤ 18 years undergoing elective surgery with general anesthesia. Patients were divided into inhalation and intravenous induction groups. LUS was performed before and after anesthesia induction to assess lung aeration. The primary endpoint was the global LUS score post-induction, with secondary endpoints including the incidence and distribution of atelectasis.

**Results:**

Of the 326 patients included, 65% underwent inhalation induction and 35% intravenous induction. The global LUS score was significantly higher in the inhalation group (12.0 vs. 4.0, *p* < 0.001). After propensity score matching (for age, presence of upper respiratory tract infection, duration of induction, and PEEP levels at induction), average treatment effect (ATE) of mask induction was 5.89 (95% CI, 3.21–8.58; *p* < 0.001) point on LUS global score and a coefficient of 0.35 (OR 1.41) for atelectasis.

**Discussion:**

Inhalation induction is associated with a higher incidence of atelectasis in pediatric patients also when we adjusted for clinically relevant covariates.

**Trial registration:**

ClinicalTrials.gov identifier: NCT06069414.

## Introduction

One established complication of general anesthesia in children is atelectasis [1]. A wide range in frequency of atelectasis has been reported in the literature in children worldwide depending on the type of surgery. In a CT-based study in 1999, Serafini described 100% of atelectasis 5 min after induction in a series of 10 pediatric patients under general anesthesia [2]. Lutterbey [3] then described atelectasis through magnetic resonance in 46 children, with a range spanning from 42 up to 80% after induction. In a study involving 40 children, Song reported a frequency of atelectasis ranging from 45 to 60% in the first ultrasound scan after induction [4].

Many factors can influence atelectasis formation during anesthesia in the pediatric population: the loss of muscular tone can reduce functional residual capacity, through both the elevation of the diaphragm induced by the abdominal organs, which is more pronounced in children given the relatively big dimensions of spleen and liver, and through the high chest wall compliance (Ccw), which is higher than lung compliance in the early stages of life [5] and cannot contrast the inward recoil of the lung. Also, the common use of high oxygen (O_2_) concentrations or choice of inadequate positive end-expiratory pressure (PEEP) levels to counteract the tendency to collapse [6] can be a co-factor in atelectasis formation.

Atelectasis results in increased intrapulmonary shunting, intra- and post-operative de-oxygenation, pneumonia [7], and post-operative pulmonary complications (PPCs) [8].

Lung ultrasound (LUS), is a “point-of-care” diagnostic and an advanced monitoring tools, non-invasive, repeatable, and environmentally friendly, which reduces patients’ exposure to ionizing radiation [9] as a green image modality and its adverse effects [10, 11].

Inducing general anesthesia in children can involve an inhalation technique, using high fresh gas flow (FGF) plus anesthetic vapor before endotracheal intubation (inhalation induction) or mask ventilation using intravenous drugs before endotracheal intubation (intravenous induction). Inhalation induction is often used to avoid venipuncture pain or facilitate vein cannulation in the pediatric population. However, inhalation induction has been associated with a higher rate of respiratory adverse events such as laryngo/bronchospasm, apnea, and upper airway obstruction [12].

Still, no study has investigated the role of inhalation vs intravenous anesthesia induction and the development of atelectasis right after anesthesia induction.

Therefore, we aimed to investigate the effect of inhalation vs intravenous technique on atelectasis occurrence, detected with lung ultrasound.

## Methods

### Ethics

This prospective observational study was conducted at a tertiary-level pediatric hospital in Milan, Italy. After approval by the Institutional Review Board (2022/ST/147), the study was registered on ClinicalTrials.gov (NCT06069414) and conducted according to Good Clinical Practice guidelines and the Declaration of Helsinki in compliance with the European General Data Protection Regulation (GDPR) 2016/679. The Reporting of Observational Studies in Epidemiology (STROBE) guidelines were followed.

### Population

Inclusion criteria were as follows: age ≤ 18 years, elective surgery under general anesthesia, and the child’s and their parents’ willingness to participate. Exclusion criteria were as follows: American Society of Anesthesiologists (ASA) physical status III–VI, neuromuscular disease, chronic pulmonary disease, cardiopathy, thoracic cage malformations, chronic home ventilation (either invasive or non-invasive), positive history of foreign body inhalation, acute or recent (previous month) lower respiratory tract infections.

### Anesthesia induction plane

The anesthesiologists in charge chose inhalational or intravenous anesthesia induction, involving in the decision also the patient and/or the caregivers as per institutional protocol. A standard monitoring was applied: electrocardiogram (EKG), peripheral oxyhemoglobin saturation (SpO_2_), and non-invasive blood pressure (NIBP).

Inhalation induction was performed with increasing concentrations of sevoflurane in air/oxygen up to loss of eyelash reflex. After peripheral vein cannulation, sevoflurane was discontinued, and the patients received 3 mg/kg of propofol and 2 mcg/kg of fentanyl intravenously. After the loss of the respiratory drive, they were ventilated via face mask with positive pressure until the airway was secured with endotracheal tube (ET) or laryngeal mask airway (LMA). If ET was planned, they also received a dose of non-depolarizing neuromuscular blocking agents (rocuronium 0.6 mg/kg). Intravenous induction was performed with 3 mg/kg of propofol and 2 mcg/kg of fentanyl, requiring positive pressure ventilation by mask with no period of spontaneous breathing. If ET was planned, they also received a dose of non-depolarizing neuromuscular blocking agents (rocuronium 0.6 mg/kg).

Per institutional protocol, FiO_2_ was maintained under 50% during induction in both groups.

All cases were managed with the same anesthesia machine, the Maquet Flow-I (Maquet, Solna, Sweden).

In both groups, the absence of air leak through the mask was confirmed by the stability of inspiratory and expiratory tidal volumes, and a positive end-expiratory pressure (PEEP) was added in the induction phase via the adjustable pressure limiting valve (APL) in the anesthesia machine, and PEEP value, confirmed on the ventilator’s monitor second by second, was recorded. After ET or LMA placement, all patients were immediately started on mechanical ventilation with the same ventilation setting: volume-controlled ventilation (VCV), tidal volume 8 ml/kg (ideal body weight), inspiratory-to-expiratory ratio 1:2, PEEP 5 cmH2O, respiratory rate adjusted for age.

### Lung ultrasonography

**Fig. 1:**
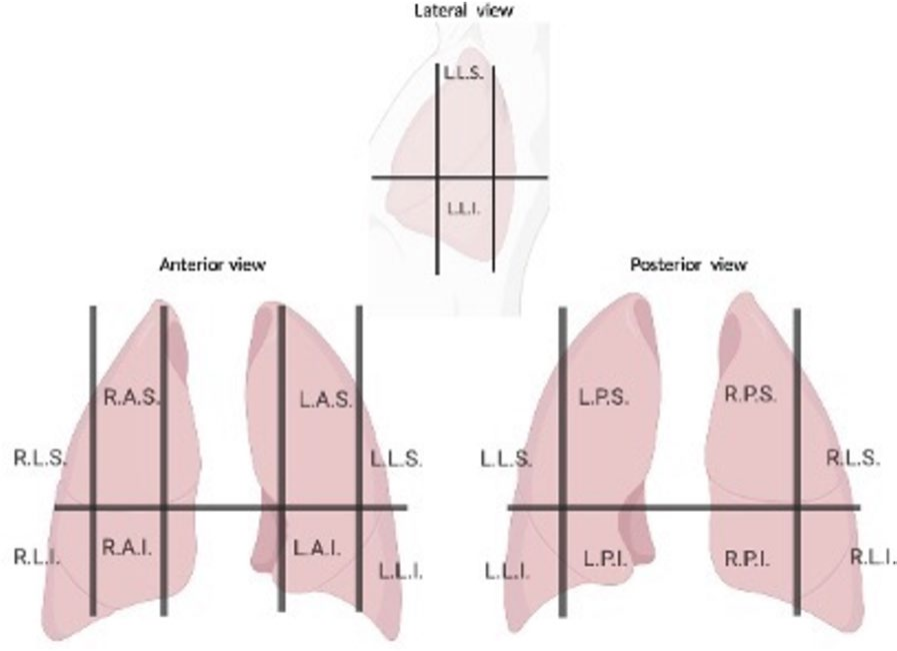
Lung areas considered: right anterior superior (RAS), right anterior inferior (RAI), left anterior superior (LAS) and left anterior inferior (LAI), right lateral superior (RLS), right lateral inferior (RLI), left lateral superior (LLS) and left lateral inferior (LLI), right posterior superior (RPS), right posterior inferior (RPI), left posterior superior (LPS) and left posterior inferior (LPI)

**Fig. 2:**
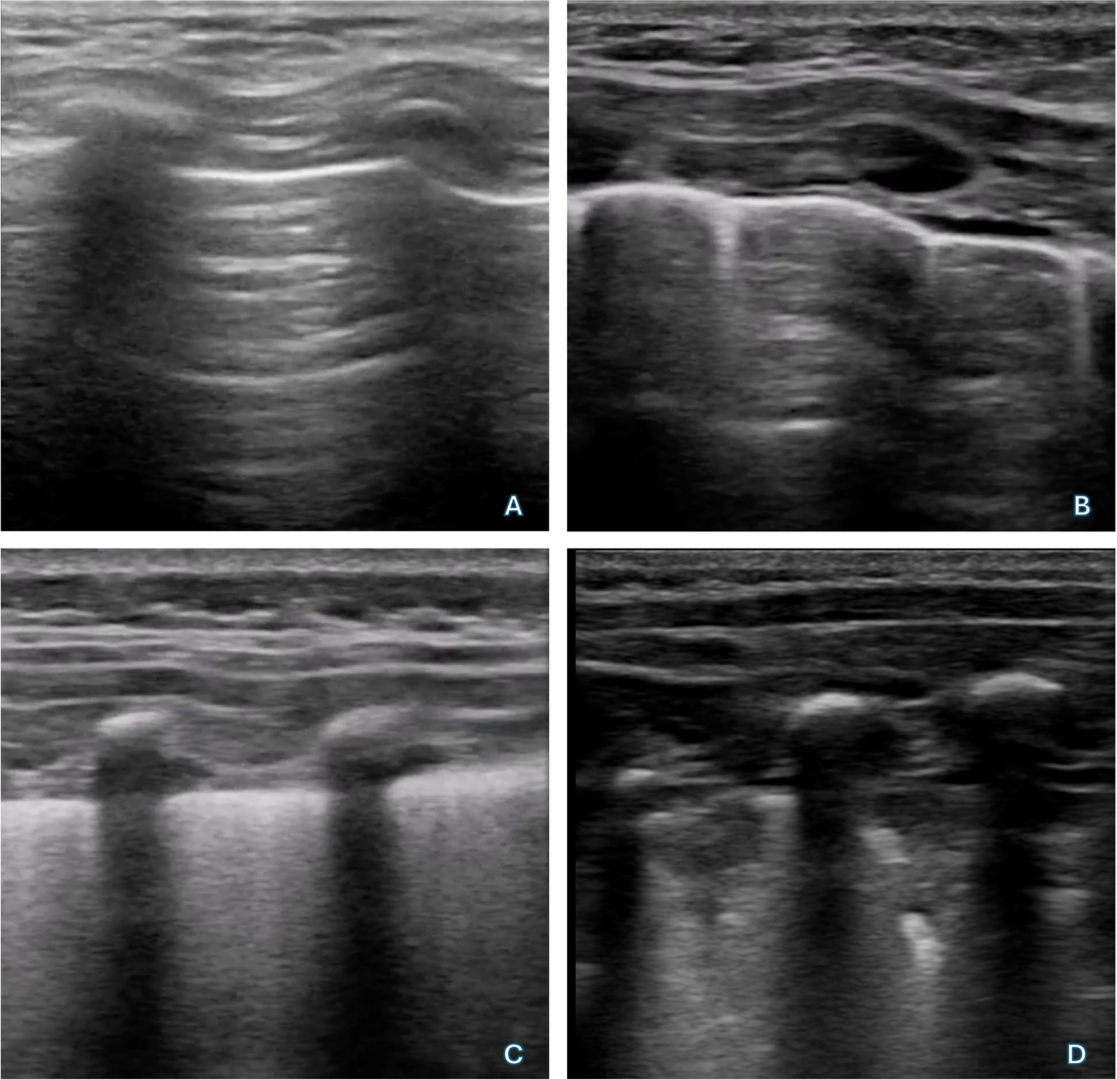
Ultrasound classification. **A** Normal aeration, A-lines or less than 2 B-lines with lung sliding [score 0]; **B**: mild alveolo-interstitial pattern—3 or more well-spaced B-lines with lung sliding [score 1]; **C** severe alveolo-interstitial pattern, represented by multiple, crowded, and coalescent B-lines occupying more than 50% of scanned intercostal space (i.e., “white” lung) with lung sliding [score 2]; **D** severe loss of aeration—tissue-like pattern or consolidation [score 3]

Before and after anesthesia induction, all patients underwent a lung ultrasound exam with a high-frequency (12–3 MHz) linear probe (Affiniti 70, Philips, Amsterdam, Netherlands) by two anesthesiologists together (A.C. and G.R.), both of whom have more than 5 years’ experience in lung ultrasonography in the operating room (OR). Video clips of the lung areas were stored and subsequently reviewed by A.C. and G.R. separately and graded. The anterior, lateral, and posterior regions of the chest were examined dividing each hemi-thorax in 6 zones as described by a recent consensus [13] (Fig. 1). For each zone, a score from 0 to 3 was assigned according to the following classification:

- Normal aeration—A-lines or less than 2 B-lines with lung sliding [score 0]
- Mild alveolo-interstitial pattern—3 or more well-spaced B-lines with lung sliding [score 1]
- Severe alveolo-interstitial pattern, represented by multiple, crowded, and coalescent B-lines occupying more than 50% of scanned intercostal space (i.e., “white” lung) with lung sliding [score 2]
- Severe loss of aeration—tissue-like pattern or consolidation [score 3] (Fig. 2)

In the calculations, the sum was considered to obtain a global score.

We defined atelectasis (either < 1 cm or > 1 cm) as the presence of subpleural consolidations and the absence of lung sliding and consolidative profile without an air bronchogram. Posterior areas were scanned rolling the patient briefly on the side. LUS scan after induction was performed in the first minute after definitive airway management. Pre-induction and post-induction scans were performed in the same position and with the same order. All patients were also scanned at the end of surgery, prior to switching the patient to spontaneous ventilation and extubation. This final scan was also performed in the same position and with the same lung area order as the two at the beginning.

### Endpoints

The primary endpoint of the study was to investigate if the global LUS score of patients induced with sevoflurane was higher than in the intravenous group; secondary endpoint was to investigate if atelectasis was higher in the inhalation induction group. Tertiary endpoint was to investigate whether the occurrence of atelectasis was related to other factors, i.e., presence of upper respiratory tract infection (URTI), FiO_2_ level at induction, length of induction.

### Sample size and power analysis

Referring to the previously published study of Lutterbey [3], which described an incidence of atelectasis of 42% in spontaneously breathing patients and of 80% in patients ventilated with positive pressure, 150 patients were considered necessary to reject the null hypothesis that the induction techniques are equal in determining atelectasis, with an alpha error of 1% and power of 95%, and including a potential drop-out rate of 20%.

### Data collections and statistical analysis

We collected the following data from each patient: age (months), weight (kilograms), sex, presence of upper respiratory tract infection (URTI, defined as signs of runny nose, cough, associated or not with fever [14]), type of airway management device used (ET versus LMA), FiO_2_ at induction, induction duration (minutes), positive end-expiratory pressure (PEEP) level (cmH_2_0) applied at induction with the APL in the anesthesia machine, use of neuromuscular blockade. Age was also divided into 6 categories, according to the definition of the American Academy of Pediatrics (AAP) [15]: newborn (from birth to <  = 1 month), infant (> 1 month and <  = 12 months), toddler (< 12 and <  = 24 months), early childhood (> 24 months and <  = 60 months) and middle childhood (60–132 months), and adolescence (> 132 months). Induction duration was measured from the start of sevoflurane administration via mask to the definitive airway (ET or LMA) placement in the inhalation group and from the injection of the intravenous anesthetic to the definitive airway (ET or LMA) placement in the intravenous group. Results are expressed as mean ± SD or median (IQR 25–75%) as appropriate in case of continuous variables and as *n*(%) for categorical variables.

Generalized linear models (GLM) were applied to study the effect of inhalation induction, presence of URTI, age, length of induction, and PEEP on the LUS scores between inhalation and intravenous induction.

Similarly, a GLM was applied to study the effect of inhalation induction on atelectasis after induction including in the model the aforementioned covariates.

As the population of patients induced intravenously differed by age from that induced with mask, and age is a confounder on the outcomes as it could be itself the cause of atelectasis, we also used propensity score matching to estimate the average treatment effect (ATE) of inhalation induction on global LUS score sum and probability of atelectasis. Data were analyzed with Stata 18.0 B.E. (StataCorp LLC, USA). Two-tailed tests were used. *P* values < 0.05 were considered significant.

## Results

**Fig. 3:**
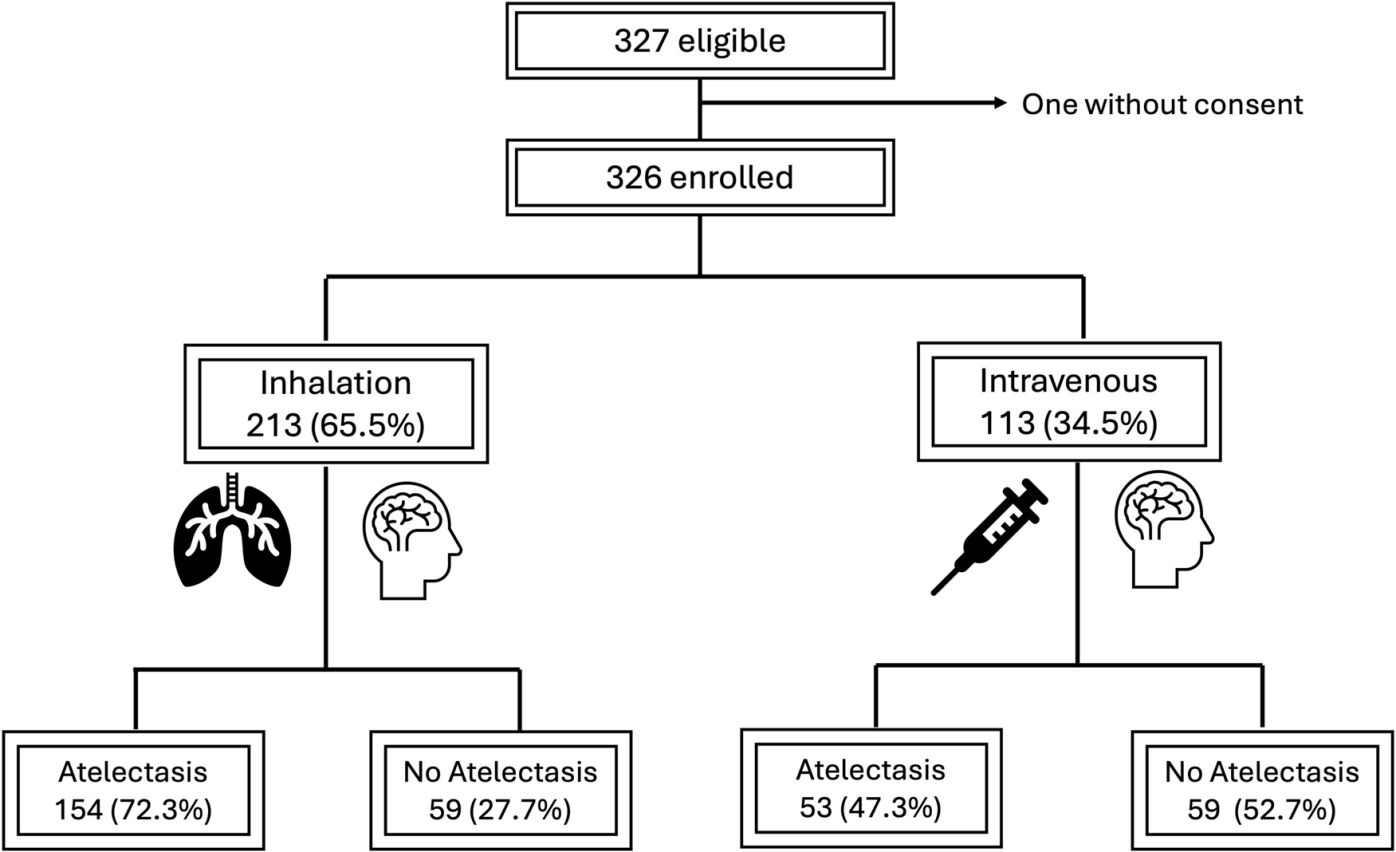
Flowchart on the enrollment and results of the study

Three hundred twenty-seven patients, from April to October 2023, were evaluated for eligibility; only one legal guardian denied the consent, leaving 326 patients for final analysis (Fig. 3).

The overall median age and median weight were respectively 50 (26–88) months and 16 (12–23) kg. The other characteristics of the study population are described in Table 1. Surgeries included routine procedures: ENT procedures (adenotomy-adenotonsillectomy), 70; laparoscopies (including herniectomy, appendectomy, varicocelectomy, nephrectomy), 55; eye surgery (strabism correction, nasolacrimal duct probe), 17; superficial pediatric surgeries (circumcision, hydrocele correction, herniectomy, cysts exeresis, hypospadias repair), 103; superficial vascular surgeries (laser), 38; orthopedic procedures (clubfoot correction, fracture repair), 43.

**Table 1:** Characteristics of patients and induction parameters

	**Total**	**Intravenous induction**	**Inhalation induction**	***p*-value**
	*N* = 326	*N* = 113	*N* = 213	
**Age, months**	50 (26–88)	106 (69–135)	38 (20–57)	< 0.001
**Weight, kg**	16 (12–23)	28 (17–40)	15 (11–18)	< 0.001
**Male sex**	202 (62%)	57(50%)	145(68%)	0.12
**ASA status I/II**	285/41	96/17	189/24	0.84
**URTI**	28 (9%)	5 (5%)	23 (11%)	0.055
**Induction duration, minutes**	7 ± 3	6 ± 2	8 ± 3	< 0.001
**PEEP at induction, cmH**_**2**_**0**	5 ± 1	4 ± 1	5 ± 2	0.14
**FiO**_**2**_** at induction**	40 ± 4	40 ± 2	40 ± 5	0.71
**Anesthesia duration, min**	50.0 (40.0–70.0)	60.0 (40.0–94.0)	50.0 (40.0–60.0)	0.014

*URTI* upper respiratory tract infection, *PEEP* positive end-expiratory pressure, *FiO*_*2*_ inspired oxygen fraction, *HR* heart rate, *RR* respiratory rate. Data are presented as mean ± SD or median (IQR) as appropriate in case of continuous variables

Before anesthesia induction, all patients showed a normal A pattern (A-line plus sliding) in the 12 lung regions, except for two patients, showing a moderate loss of aeration (score 1) in the posterior-superior area.

A total of 213 (65%) underwent anesthesia induction via inhalation technique, while 113 (35%) had an intravenous induction. One of the two patients presenting abnormal findings before induction received inhalation induction and the other one intravenous induction. No patient experienced respiratory adverse events (laryngospasm, bronchospasm, desaturation ≤ 95%, coughing, airway obstruction) at induction.

After anesthesia induction, 207 children (65%) presented atelectasis in at least one of the lung areas; of these, 154 had undergone inhalation induction and 53 intravenous induction (Pearson’s chi-squared, 20.54; *p* < 0.01). Atelectasis was significantly associated with age category (Pearson’s chi-squared = 11.82, *p* = 0.037). Regional LUS showed that atelectasis affected mostly posterior and superior areas of the lung (Table 2).

**Table 2:** Regional distribution of atelectasis after induction (Wilcoxon rank-sum test)

	**Intravenous**	**Inhalatory**	***p*-value**
	*N* = 112	*N* = 213	
**R.A.S**	0 ( 0.0%)	3 (1.4%)	0.21
**R.A.I**	0 (0.0%)	1 (0.5%)	0.47
**R.L.S**	1 (0.9%)	3 (1.4%)	0.72
**R.L.I**	3 (2.8%)	3 (1.4%)	0.38
**R.P.S**	17 (16.0%)	92 (43.6%)	< 0.001
**R.P.I**	28 (26.4%)	81 (38.4%)	0.034
**L.A.S**	1 (0.9%)	1 (0.5%)	0.62
**L.A.I**	0 (0.0%)	1 (0.5%)	0.47
**L.L.S**	3 (2.8%)	6 (2.8%)	0.98
**L.L.I**	2 (1.8%)	1 (0.5%)	0.23
**L.P.S**	22 (21.0%)	96 (46%)	< 0.001
**L.P.I**	39 (36.8%)	95 (45%)	0.31

*RAS* right anterior superior, *RAI* right anterior inferior, *LAS* left anterior superior and *LAI* left anterior inferior, *RLS* right lateral superior, *RLI* right lateral inferior *LLS* left lateral superior and *LLI* left lateral inferior, *RPS* right posterior superior, *RPI* right posterior inferior, *LPS* left posterior superior and *LPI* left posterior inferior

The global LUS score was significantly different between inhalation and intravenous induction: 12.0 (6.0–17.0) for inhalation and 4.0 (0.0–12.0) for intravenous induction (*p* < 0.001).

A GLM was conducted on the outcome “LUS global sum after induction” including inhalation technique, age, presence of URTI, Peep level in the anesthesia machine at induction, use of neuromuscular blockade, and duration of induction as covariates. It showed a coefficient of 5.05 (95% CI, 1.37; 8.73; *p* = 0.007) for inhalation induction. GLM conducted for the outcome “atelectasis” showed a coefficient of 0.28 (95% CI, 0.10; 0.45; *p* = 0.002) for inhalation induction, equivalent to an OR of 1.32.

After propensity score matching (where match was performed on age, presence of URTI, duration of induction, and PEEP levels at induction), average treatment effect (ATE) of mask induction on atelectasis and global LUS score was calculated. ATE for mask on atelectasis occurrence was 0.35 (95% CI, 0.14–0.55; *p* = 0.001) which translates into an odds ratio of 1.41 for atelectasis occurrence when inhalation induction and given the above-mentioned propensity score match. The coefficient of ATE for mask induction on global LUS score was 5.89 (95% CI, 3.21–8.58; *p* < 0.001).

At the end of surgery, the number of atelectatic areas was still significantly associated with inhalation induction (*p* = 0.032).

## Discussion

The main findings of the present study are as follows: (1) the global LUS scores of patients induced with inhalation technique are higher and the most affected regions are the posterior and superior ones, (2) atelectasis has higher incidence in patients induced with inhalation technique, (3) atelectasis is higher in this group even when adjusting for the age of the patients, the presence of URTI, and the slightly longer duration of induction.

Induction via inhalation technique is often required in pediatric anesthesia to avoid venipuncture pain and facilitate peripheral venous cannulation. During this time, patients breathe spontaneously in the anesthesia circuit, and with loss of consciousness and reduced muscular tone, we hypothesized there is a potential for atelectasis formation.

Suppose every anesthesia is at risk for atelectasis formation due to different mechanisms (compression, impairment of surfactant function, absorption of gases [6]). This is particularly true in the pediatric population, where functional residual capacity and closing capacity are in an unfavorable balance [6, 16, 17]. We therefore postulated that a period of spontaneous breathing with increasingly reduced muscular tone due to anesthesia deepening could pose pediatric patients at an increased risk of atelectasis formation.

Physiological differences in children’s chest wall compliance (Cw) compared to adults may play a major role in their higher predisposition to atelectasis formation during anesthesia induction. Indeed, children have a highly compliant chest wall with a reduced ability to counteract the elastic recoil of the lung tissue [18–20]. The developmental increases in chest wall stiffness over the first years of life may contribute to the maintenance of a higher resting lung volume, thus reducing atelectasis formation in older children compared to newborns and infants.

Inhalation induction is more used in smaller children, because it is often more difficult, or less accepted, to institute a peripheral venous access in these awake patients. Inhalation induction, also, through the maintenance of spontaneous breathing, is extremely useful when a difficult airway is suspected. Atelectasis could be explained by the different structures of the rib cage in smaller children than in bigger ones: the smaller the baby, the more compliant the thoracic cage and the bigger the collapsing forces on the lung [19, 20].

Notably, the effect of inhalation induction on atelectasis formation remained significant after propensity score matching, and the global LUS score at induction was significantly higher in children anesthetized with inhalation technique after the same correction. A role in atelectasis formation could also be attributed to sevoflurane, which is used in the inhalation group. Although sevoflurane is a non irritating agent which has dramatically reduced the incidence of laryngospasm at induction [21], still an animal study reported a potential effect of sevoflurane on surfactant function (i.e., changes in surfactant composition and viscosity properties) which could potentially impair lung mechanics and promote the alveolar collapse [22].

Previous papers have highlighted the high incidence of atelectasis in pediatric patients. Acosta [23] showed atelectasis in 14/15 children anesthetized with sevoflurane for magnetic resonance and spontaneously breathing. More recently, Kim studied the effect of different FiO_2_s at induction on atelectasis formation and found atelectatic areas in 51/52 patients enrolled.

High-inspired fractions of oxygen (FiO_2_) are often employed to pre-oxygenate patients during induction, and this factor too can promote atelectasis [6] due to absorption [18]. Pediatric patients could be considered a risk population for hypoxia, and it is possible that high FiO_2_s are employed in different institutions per protocol. Although it is true that children do show a shorter duration of non-hypoxic apnea time [24], our institutional protocol proposes FiO_2_s lower than 50% at induction in healthy children to avoid both the potential side effects of oxygen at the cellular level [25] and the detrimental effect of oxygen on atelectasis formation [26]. FiO_2_ is raised if a difficult airway is suspected or if any complication ensues. This approach proved safe in our cohort, as demonstrated by the absence of hypoxic events, but certainly cannot be generalized for any kind of pediatric patient and for any level of experience of the operators.

The presence of upper respiratory tract infections has been considered a covariate in the analysis as it is a potential confounder: the presence of secretions could be an additive factor in the formation of atelectasis if they accumulate in distal bronchioles.

However, their presence did not significantly modify the effect of mask induction on the outcome, reinforcing that the findings of peripheral atelectasis are possibly related to the kind of anesthesia induction.

Inhalation induction has been associated historically with a higher risk of respiratory complications [14, 27, 28]. Although we did not record any respiratory complications in our patients, the results of our study could confirm the role of inhalation induction in association with respiratory adverse events.

Of note, a moderate level of PEEP was added in both groups, by modulating the APL valve. The efficiency of the PEEP at induction is guaranteed by adequate mask seal and confirmed by the ventilator. PEEP at induction shows a protective effect on atelectasis formation in our cohort, as expected.

Our work shows that the posterior lung areas are the most affected by atelectasis, as expected, since induction happened in the supine position in all patients. Dependent areas are at risk due to gravity [6]; a work reported that in spontaneously breathing normal subjects receiving volatile anesthesia, the activity of parasternal muscles is abolished, and phasic expiratory activity in abdominal and lateral rib cage muscles is enhanced, contributing to caudad-dependent atelectasis [29]. Interestingly, we found that posterior-superior lung areas showed the highest incidence of atelectasis among all lung areas, in opposition to previous work conducted in adults [30]. Our group recently described a similar phenomenon in patients affected by bronchiolitis [31] who are, however, generally smaller in age and weight.

LUS is easy to perform, sensitive, and specific for the purpose we described. It may be however not always feasible or available, and other methods could be employed to detect the presence of atelectasis. Recently, a test was proposed (the “Air-Test”) [32] based on the relationship between FiO_2_ and SpO_2_, which has shown very high sensitivity and specificity for the detection of atelectasis.

Our paper reinforces the knowledge that atelectasis formation is a relevant problem in pediatric anesthesia and shows a very high incidence. As a response to the development of atelectasis, several methods have been proposed, such as PEEP increase [33] or recruitment maneuvers [34–37]. Again, there is no single answer for all situations as the best solution. LUS can be used also to recheck lung aeration after corrective maneuvers have been put in place.

### Limitations and strengths

We acknowledge that the non-randomized design of the study represents a limitation because the two groups eventually differed under different aspects. We believed however that for clinical and ethical reasons (i.e., difficult intravenous access, children who were not compliant in getting peripheral intravenous placement, pain experienced by children), patients could not be randomized. In order to mitigate potential confounders, a propensity score method was employed. This statistical technique [38] was used to account for the probability of receiving mask induction given baseline covariates and thus interpret results more realistically. Another limit of the study design is that the investigators performing the LUS were not blinded to the treatment group.

On the other side, we believe that this study has several strengths, such as the relevant number of patients enrolled covering all the pediatric ages and the ultrasound being carried on by the same two operators to reduce operator dependency of the results. Also, a thorough lung scan was performed in all patients, which included all lung areas with mobilization of the patient in order to achieve the best possible view also in the posterior fields.

## Conclusions

In conclusion, after anesthesia inhalation induction, a great percentage of atelectasis was detected in the lung of children monitored with LUS. Understanding how and when and what strategies could be implemented to reduce atelectasis is of paramount importance. In our court of children, LUS was an easy and safe and advanced monitoring tool. Randomized controlled trials are needed to confirm our hypothesis and study whether or not atelectasis correlates with clinical consequences in the post-operative period.

## Competing Interests

The authors declare no competing interests.

## Data Availability

No datasets were generated or analysed during the current study.
